# Strategies for Safe Implantation and Effective Performance of Single-Chamber and Dual-Chamber Leadless Pacemakers

**DOI:** 10.3390/jcm12072454

**Published:** 2023-03-23

**Authors:** Fei Tong, Zhijun Sun

**Affiliations:** Department of Cardiology, Shengjing Hospital of China Medical University, Shenyang 110004, China; tongfeimed@163.com

**Keywords:** leadless single-chamber pacemaker, complication, atrioventricular synchrony, leadless dual-chamber pacemaker, strategy selection

## Abstract

Leadless pacemakers (LPMs) have emerged as an alternative to conventional transvenous pacemakers to eliminate the complications associated with leads and subcutaneous pockets. However, LPMs still present with complications, such as cardiac perforation, dislodgment, vascular complications, infection, and tricuspid valve regurgitation. Furthermore, the efficacy of the leadless VDD LPMs is influenced by the unachievable 100% atrioventricular synchrony. In this article, we review the available data on the strategy selection, including appropriate patient selection, procedure techniques, device design, and post-implant programming, to minimize the complication rate and maximize the efficacy, and we summarize the clinical settings in which a choice must be made between VVI LPMs, VDD LPMs, or conventional transvenous pacemakers. In addition, we provide an outlook for the technology for the realization of true dual-chamber leadless and battery-less pacemakers.

## 1. Introduction

Conventional transvenous pacemakers (TPMs) have been the cornerstone of the treatment of bradyarrhythmias. Researchers have estimated that more than one million devices have been implanted annually in recent years [1]. Despite numerous technological advances since the introduction of TPMs, lead- and pocket-related complications are still common. Acute complications involving pneumothorax, cardiac perforation, lead dislodgment, pocket infection, or hematoma occur in 12% of patients [2], and chronic complications such as lead malfunction, lead-related endovascular infection, and tricuspid valve (TV) dysfunction occur at rates of 2.5–5.5%, 0.5–1.3% and 14.5%, respectively, in those who have received TPM implantation [3,4,5,6]. With technological advances in device miniaturization, communication, and battery longevity, leadless pacemakers (LPMs) have emerged as an alternative to TPMs to eliminate the complications associated with leads and subcutaneous pockets. However, the LPM usage was restricted by its indication area (single-chamber only) and specific complications, with a short-term complication rate of 4–6.7% [7,8] and a chronic complication rate of 4.6–6.6% [9,10]. Many strategies, including appropriate patient selection, procedure techniques, device design and postimplant programming, have been developed to overcome complicated situations. In this review, we summarize the safety and efficacy of the currently available LPMs, and we discuss strategies to minimize the complication rate and maximize the efficacy, providing an outlook for the technology for the realization of true dual-chamber leadless and battery-less pacemakers.

## 2. Leadless Ventricular Pacemakers

### 2.1. Brief History and Current State of Two Leadless Systems

Nanostim (St.Jude, Saint Paul, MN, USA), as the first commercially available LPM capable of the VVI(R) pacing mode, was launched in 2012; however, the use of Nanostim implantation was discontinued due to the detachment of the docking button and premature battery failures, which occurred at two years after implantation [11]. The Aveir VR (Abbott, Abbott Park, IL, USA) by Abbott, which is an improvement of the Nanostim LPM, received Food and Drug Administration (FDA) approval on April 2022, and it could provide an expandable platform to support dual-chamber pacing once approved by the FDA [12].

The Micra VR (Medtronic, Minneapolis, MN, USA), which is now widely used all over the world, was first implanted in 2013 and it obtained FDA approval in 2016. The main indications for the Micra VR implantation are atrial fibrillation (AF) with slow ventricular response, as well as non-AF with low anticipated ventricular pacing, such as transient atrioventricular (AV) block and sinus node dysfunction [13,14]. The Micra AV (Medtronic) is the only currently available LPM that is capable of delivering the VDD pacing mode [15,16]. With an identical mass, appearance, design, and implant procedure to the Micra VR, the novel algorithm of the Micra AV discerns the signal of atrial mechanical contraction through the intracardiac accelerometer from the device in the right ventricle (RV) and fulfills AV synchrony. Limited by the mechanism of accelerometer-based atrial sensing rather than electric atrial sensing, and the absence of a pacing device in the right atrial (RA), the Micra AV is not suitable for those indicated for conventional DDD-TPMs, such as those with sick sinus syndrome and poor atrial contraction.

Elderly or malnourished patients with high infectious risk are prone to choosing LPMs [17,18]. Patients on haemodialysis would benefit from LPM implantation because it spares the subclavian and superior cava veins for dialysis treatment. The obstruction of the venous route used for TPM and potential pocket issues (e.g., in the case of dementia) are the indications for LPM [17]. Apart from patients with clinical frailty, younger patients also choose LPMs out of esthetical or active lifestyle concerns [19]. However, the scant clinical data regarding the end-of-life strategy and the possibility of implanting two or more LPMs in the same patient limit the routine use of LPMs in patients with a life expectancy of >20 years [17].

### 2.2. Evaluation of Clinical Performance and Recommendation of Strategies

The success rate of LPM implantation was extraordinarily high for the Micra VR, with 99.2% (719/725) in the Micra Investigational Device Exemption (IDE) trial [20] and 99.1% (1801/1817) in a real-world setting [21]. The success rate was 98% (196/200) for the Aveir VR in the LEADLESS II phase 2 IDE study. The mean pacing threshold and R-wave amplitude of the Micra VR were 0.66 ± 0.55 V at 0.24 ms and 11.1 ± 5.2 mV at implantation, and the electrical parameters remained stable during 18 months of follow-up [21]. A total of 196 patients were successfully implanted with the Aveir VR. For 95.9% of these patients, the pacing thresholds were less than 2.0 V at 0.4 ms and the R waves were greater than 5.0 mV at the 6 week follow-up [12]. However, no clear consensus has been determined in terms of the complication rate of LPMs when compared to TPMs. The Micra VR was associated with 48% and 63% lower risks of major complications than those of a historical TPM cohort in the Micra IDE trial [20] and the Micra Post-Approval Registry (PAR) [21], respectively, during the 12 month follow-up period. The analyses were based on the comparison with a historical TPM cohort and a long-term follow-up period [20,21]. However, a meta-analysis on four studies showed no difference in the incidences of any complications between LPMs and TPMs [22]. Moreover, in a prospective analysis, no significant difference at an almost 2 year complication rate was observed between LPMs and TPMs [23], which could be because the contemporary complication rate of TPMs is significantly lower than the historical one as a result of standard implantation procedures and improved techniques. However, a contemporary prospective propensity-matched analysis also demonstrated that the rate of complication in a TPM cohort was 4.9% vs. 0.9% in a LPM cohort, during 800 days of follow-up and after excluding the pacemaker advisory-related complications [24]. Furthermore, in real-world practice, Micra implantation (*n* = 16,825) is associated with a lower complication rate of 8.6%, which is lower than the 11.2% of contemporary TPM implantation (*n* = 564,100) [25]. A continuous enrollment study and contemporaneous comparison of the Micra and TPMs in the Micra Coverage with Evidence Development (CED) study observed that the Micra implantation was associated with 23% fewer and 31% fewer complications compared with TPMs over 6 months [26] and 2 years [9], respectively, indicating that the fewer LPM complications were due to a time-dependent effect, which was also manifested by the improved LPM complication rates from the 6 month follow-up to the 2 year follow-up, and the similar 30 day adjusted complication rate of LPMs to that of TPMs [26]. A large and real-world analysis of the national database from the United States showed that, overall, the unadjusted in-hospital complication rate of 16% for LPMs was higher than that of the 6.4% for TPMs; however, it should be noted that the patients with LPMs in this analysis were older and had more sepsis, chronic kidney disease, heart failure and malnutrition [18]. Transvenous lead- and subcutaneous-pocket-related complications account for most of these TPM complications [3,4], and they take time to occur. In contrast, the more frequent incidences of cardiac perforation and pericardial effusion in LPMs than TPMs and the relatively high incidence of vascular complications in LPMs are short-term complications, both of which form a non-conclusive picture of the complications encountered with LPMs vs. TPMs. The discrepancies of the complication percentages between studies are partly because some studies only report complications requiring reinterventions [24], while some studies describe all complications [25].

#### 2.2.1. Cardiac Perforation and Pericardial Effusion

The overall rate of cardiac perforation and pericardial effusion in the Micra IDE, Micra PAR, and Micra Continued Access study was 1.1% (32/2817) [27]. The risk of pericardial effusion after the implantation of Micra decreased from 1.8% to 0.8% over time [27]. The rate of pericardial effusion was 0.8% in the Micra CED study. The Manufacturer and User Facility Device Experience (MAUDE) report also estimated a 1% incidence of cardiac tamponade, and more than three times the cardiac tamponade of the Micra compared with the TPM ventricular lead [28]. In a real-world setting, 1.3% of LPM recipients suffered from cardiac effusion or perforation [18], and the figure was significantly higher than that of TPM recipients [25]. Similarly, as for Aveir VR implantation, the rate of cardiac tamponade was 1.5% (3/200) in the LEADLESS II-phase 2 IDE study [12]. A relatively high rate of cardiac perforation following LPM implantation could be worrying; hence, we recommend measures to be taken and strategies to limit those adverse events:

Patients’ characteristics: Apart from the patients’ characteristics, such as old age, female gender, and chronic obstructive pulmonary disease (COPD) that easily develops cardiac injury [20], a low body mass index (BMI) and congestive heart failure were also identified by the PAR investigators as predictors of perforation [21]. A novel risk score model, which included similar risk factors such as age of >85 years, BMI of <20, female gender, heart failure, prior myocardial infarction, COPD, haemodialysis, and the absence of previous cardiothoracic surgery, was developed and validated to predict pericardial effusion [27]. Additional attention should be paid to the presence of the abovementioned risk factors.

**Figure 1:**
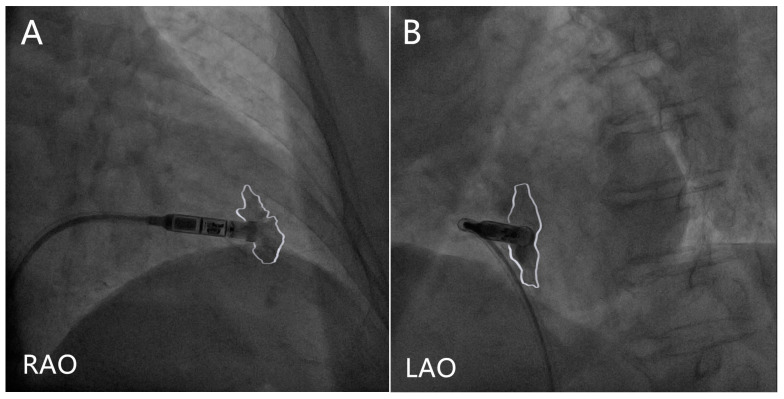
Contrast injection in right anterior oblique (RAO) and left anterior oblique (LAO) projections. The white lines delineate the contrast outlines. (**A**) The contrast seen both in front of and inferior to the tip of the delivery catheter in RAO projection suggests a non-apical location. (**B**) The contrast delineates the ventricular septum in LAO projection.

Procedure technique: The positioning of the Micra at the interventricular septum has been linked to a lower risk of cardiac perforation compared with the apex or free wall [20], and deployment in the mid-septum should be achieved [29], which leads to a narrower-paced QRS complex [30]. However, deploying a Micra near the anterior interventricular groove might increase the risk of perforation [31]. Contrast injection is recommended to ensure a mid-septal position in orthogonal fluoroscopic views prior to deployment [29], and RV trabeculation in front of and inferior to the tip of the delivery catheter suggests the septum location, as opposed to a lack of trabeculation, which suggests the apical location [29] (Figure 1). The combination of orthogonal fluoroscopic views and transthoracic echocardiography in the subxiphoid, parasternal, and apical views without contrast injection significantly reduces inadvertent non-septal implantation compared with ventriculography [32]. The pacing threshold will frequently improve over 2–3 min, and especially when the impedance suggests good myocardial apposition (>500 ohms). Therefore, it is reasonable to allow at least 3 min for the thresholds to improve before deciding on an alternate location [33]. As for the sensed R-wave, a 1.5-mV increase in R-wave amplitude after approximately 13 min since the first deployment is predictive of a reduction of pacing threshold below 1 V/0.24 ms at follow-up, which also suggests that waiting and evaluating a second electrical parameter may be capable to avoid unnecessary device repositioning in case of high pacing threshold recording at implant [34].

Device design: The tine-based fixation mechanism of the Micra permits the measurement of the electrical parameters only after deployment and active fixation into the myocardium, which inevitably increases the need for repeated attempts at deployment once the parameters are unsatisfied and there is a risk of pericardial effusion [27,35]. Different from the Micra, the contact mapping capability of the Aveir VR helps to optimally position the device before fixation [12,36], which could prevent the need for multiple attempts. Of the successful implants, 83.2% (163/196) of the Aveir VR implantation did not require repositioning in the LEADLESS II phase 2, while one deployment sufficed in only 60.0% (1583/2638) of the Micra implantations [27]. In contrast to adequate forward pressure applied to form the “goose neck” appearance of the delivery system prior to the implantation of the tine-based Micra, firm pressure is not required nor recommended and a softer touch with tissue contact is sufficient for the Aveir VR to achieve stable fixation via the slow rotation of device [36].

Rescue strategies: Unlike transvenous lead perforations, Micra perforations are often large and life-threatening. The MAUDE reports a higher rate of mortality following perforations with the Micra compared with TPMs [28]; thus, intervention that includes pericardiocentesis and surgery is required to rescue patients. In the Micra PAR, 71% (10/14) of the cardiac effusion patients required pericardiocentesis and 14% (2/14) required surgical repair [21]. Data from a real-world setting showed that 36% (82/228) of pericardial effusion patients had the need for pericardiocentesis, and 11.5% (26/228) required a thoracotomy. A higher proportion (26% (146/563)) of Micra-related perforations requiring emergency surgery was also reported in the MAUDE [37].

#### 2.2.2. Dislodgment

Micra dislodgment was rare and limited to case reports with no dislodgment in the Micra IDE trial [20]. A total of 0.06% of the implantations resulted in dislodgment in the Micra PAR [21], and 0.51% (40/7821) of the implantations resulted in dislodgment in the real-world setting, in comparison with the relatively high rate of lead dislodgment in TPMs, which ranged between 1% and 2.55% [38]. The dislodged device could migrate to a remote location, such as the pulmonary artery, causing acute respiratory failure [39], or could produce no symptoms if the LPM is wedged in a stable manner [40], or it could be stuck in the right ventricle, causing non-sustained ventricular tachycardia [41] and damage to the TV and papillary muscles. Some types of dislodgment only manifested the loss of capture without obvious dislocation [42].

Patients’ characteristics: Due to the limited amount of data, no risk factors concerning the underlying etiology for dislodgment were identified. Based on the currently available case reports, a complex heart anatomy [41,43] and myocardial fibrosis or scars in cardiac amyloidosis or ischemic cardiomyopathy might influence the engagement of the tines [41].

Procedure technique: The fixation of at least two tines is acceptable for the Micra according to the manufacturer’s training recommendation; however, several dislodgment cases met the criterion of two tines [39,44,45], which indicates that the movement of two tines by the pull-and-hold test is not a guarantee against dislodgment, and that the pull-and-hold test, per se, is not an objective evaluation of the tine movement. A stable and low pacing threshold (<1 V at 0.24 ms is ideal; however, ≤2 V at 0.24 ms is considered acceptable), high sensed R wave, high impedance, and the recorded current of the injury may indicate solid fixation. Unstable impedance by the repeated measurement could suggest an insecure connection between the device and myocardium [44]. Final implant thresholds above 2 V are not recommended [33]. In situations of multiple reposition failures, it is recommended to remove and re-flush the delivery system to clean off clots, and an effective strategy is to implant LPM in the apical position and to obtain the R-wave at approximately 10 mV and the pacing threshold far below 1V in a series of three to four interrogations [46]. This strategy can be attempted by experienced operators, and is not recommended for operators at the beginning of the learning curve in consideration of the risk of cardiac perforation [46].

Rescue strategies: A Micra introducer sheath with either a delivery catheter or steerable Agilis sheath is implemented to align with the Micra with an acute rise in capture threshold, but without obvious dislocation noted, and loop snares of different sizes (range 7–10 mm) and shape (single loop or multiple loop) with integrated protective sleeves are used to capture the proximal retrieval feature or the body of LPM if the proximal retrieval feature cannot be engaged [47]. In complex situations, such as the migration of the device to the pulmonary artery or its free movement in the RV, the two-snare technique is applied as follows: one snare captures any tine that is not engaged in the endocardium to minimize the Micra movement, and a second snare captures the retrieval feature or the body of the Micra capsule [41,42,44,48]. Additionally, a gooseneck snare from the femoral venous approach can also be used to retrieve LPM, embolizing the pulmonary artery [49].

#### 2.2.3. Vascular Complications

The Micra VR and Aveir VR are both delivered through 27F (outer diameter) introducer sheaths. Large-bore venous access for LPM implantation could induce vascular complications, such as arteriovenous fistulas (AVFs), pseudoaneurysms, bleeding and hematomas. Incidences of 0.6–1.4% for vascular complications were reported in the Micra PAR study [21] and Micra CED study [26].

Medicine preparation: The Micra VR and Aveir VR are approved for patients with AF complicated by bradycardia. The results from the Micra PAR indicated whether the intermittent interruption of anticoagulation in the perioperative setting could not significantly influence vascular-related events [50], which meant that the Micra without the interruption of anticoagulation could be performed. Although no suggestions from the guidelines are provided on the perioperative anticoagulation management in LPM procedures, continued warfarin if the international normalized ratio is <3 and the temporary interruption of new oral anticoagulants 24 h before the LPM procedure may be safe and have already been applied in clinical practice [50,51,52].

Procedure technique: A puncture site of the femoral vein just higher or at the level of insertion of the great saphenous vein is recommended to reduce the risk of AVF [29]. A puncture site below the common femoral artery or vein is a risk factor for pseudoaneurysm. In complex cases in which multiple arteries are inadvertently punctured, vascular ultrasound guidance or micropuncture techniques could be considered to avoid vascular complications [50]. More than half of the cases of haemostasis following LPM implantation were achieved using figure-of-eight sutures [53], and the application of pressure alone should be avoided for the 27F introducer sheath [29]. Surgical isolation of the common femoral vein for the purpose of sheath insertion in patients with severe obesity and vascular haemostasis and suture performed by vascular surgeon were effective and safe in LPM implantation [54].

Rescue strategies: Studies on AVF indicated that 38% of the cases of iatrogenic femoral AVF self-resolved at 1 year [55] and that iatrogenic AVFs should be repaired using a covered stent to seal the shunting of the AVF only when the shunting has hemodynamic consequences [56]. However, these AVFs were vascular access complications of percutaneous coronary interventions, in which sheath sizes of 7F or 8F were used. As for LPM implantation, in which a sheath of 27F is used, intervention or surgical repair may be a necessary choice. A stable pseudoaneurysm diameter of <2 cm can be conservatively managed with observation, and a pseudoaneurysm diameter of >2 cm can be managed with ultrasound-guided thrombin injection, surgical repair, or covered stent placement [57].

#### 2.2.4. Infection

By virtue of the elimination of surgical pockets and transvenous leads, less surface area and endovascular encapsulation, LPMs have a low incidence of infection, as no infections were identified among the 3726 patients with LPMs during the 6 month follow-up in the Micra CED study [26]. A total of 33 infections out of 726 cases was recorded in the Micra IDE trial [20]; however, none of these events were associated with device- or procedure-related infections. Procedure-related infections, including abdominal wall infections, infected groin hematomas, and sepsis, occurred at 0.17% in the Micra PAR [21]; however, none of these infection cases required device removal. As for the Aveir VR, no device-related infections have been reported so far [12]. LPMs have been shown to have some unique characteristics that make them suitable for patients with high infectious risk, and in situations in which infected transvenous leads or pockets have already occurred. Suggestions according to the different scenarios are listed as follows:

Medicine preparation: Almost all the patients in an Italian clinical practice were given a prophylactic dose of antibiotics before the LPM implantation procedure without additional adverse events [36,53]; however, the specific prophylactic antibiotic usage for LPMs in the perioperative setting needs further exploration.

Application strategies: No evidence of recurrent infection was found following the LPM implantation at or after the infected TPM removal [35,58,59,60]. In the case of lead- or pocket-related infections, the LPM implantation could even be performed before the extraction of an infected TPM for pacemaker-dependent patients, without the occurrence of reinfection [60,61]. In cases of bacteremia or endocarditis, it is recommended that the LPM not be implanted until the blood cultures turn negative, and for pacemaker-dependent patients, temporary pacing through jugular access is the interim solution after the removal of an infected TPM and prior to LPM implantation.

#### 2.2.5. Tricuspid Valve Regurgitation

The development of tricuspid valve regurgitation (TR) after TPM implantation is primarily caused by the mechanical interference of the ventricular leads with the TV and its sub-valvular apparatus [62]. Despite the absence of leads crossing the TV, LPMs with lengths of 42 mm for the Nanostim, and with lengths of 25.9 mm for the Micra, still have the potential to interact with the valvular apparatus. The aggravation of TR was observed in 12% of patients with LPMs during the 48 month follow-up when compared with 9% of patients with TPMs [63]. However, an age- and sex-matched analysis showed a higher increase in the TR severity in patients with TPMs than in those with LPMs [64], and the LPMs had the advantage of reducing the TR effective regurgitant orifice area, compared with conventional leads 1 month after the device implantation [65]. The TR increased 12 months after the LPM implantation; however, it was comparable to that of TPMs [66]. The conclusion concerning TR following LPM implantation is controversial, which is possibly because of the differences in the definition of TR, the follow-up period, and the proportion of the Nanostim, with a greater length than the Micra. To minimize the potential influence of LPMs on the TV, suggestions are provided for consideration.

Procedure technique: A septal implantation of the Micra is recommended to reduce the risk of cardiac perforation, which may not be a risk factor for the worsening of TR. Apical septal implantation is considered desirable to avoid the entrapment of the docking button or proximal retrieval feature within the tricuspid valve apparatus. However, a basal implantation site close to the TV annulus should be avoided to minimize mechanical interference with the valvular apparatus [66]. Despite being designed 10% shorter than its predecessor the Nanostim, the Aveir VR, which is 38.0 mm in length, is still longer than the Micra [36], and therefore the Aveir VR implantation site should maintain more of a distance from the TV and anterior interventricular groove if possible. Physicians should balance the long-term benefit of the TR severity with the short-term risk of pericardial effusion.

### 2.3. Gaps in Experience

Worldwide experience demonstrated that the early retrieval of Micra (median 46 days, range 1–95 days) was feasible and can be accomplished with low risk of serious complications, such as cardiac perforation, and device embolization [47]. However, the long-term experience with Micra retrieval is limited. The Micra VR was designed with 12 years of battery life, and the battery longevity was confirmed in the real-world setting [67]. As for the Micra AV, the original design of an 11.8 year battery life (1 V, 0.24 ms) with 100% pacing was reduced to 10.5 years in the real-world setting [68]. The anticipated encapsulation of LPMs during the whole battery life could further impede the extractability of LPMs. There is no recommended treatment for LPMs after battery depletion. Physicians can either retrieve the nonfunctioning LPMs and subsequently implant a new device, or abandon the nonfunctioning LPMs and implant a new adjacent one [69]. Even though the RV can host up to three Micra devices [70], this could still lead to geometric alterations of the cardiac anatomy and have a negative impact on the ventricular volume.

## 3. Leadless AV Synchronization

### 3.1. Brief Introduction of the Algorithm of the Micra AV

**Figure 2:**
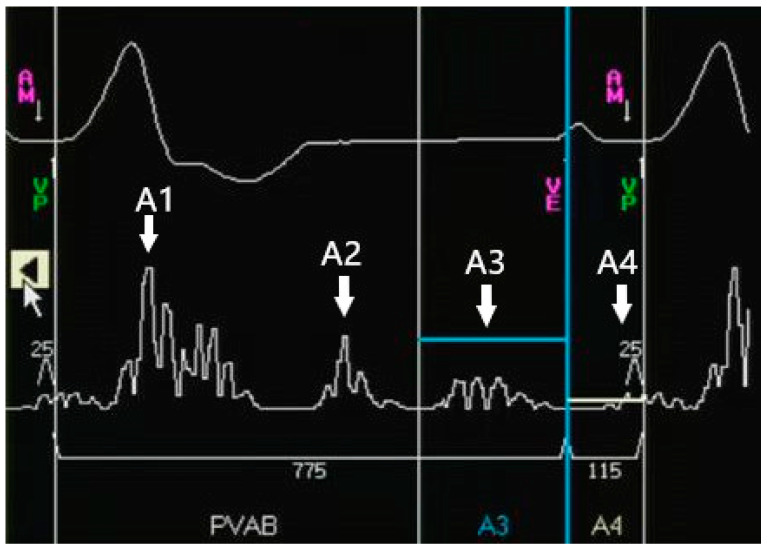
Schematic illustration and explanation of the key atrial sensing parameters. The top signal shows the electrocardiogram; the bottom signal shows the accelerometer signal.

Four distinct segments of cardiac activity are derived from the accelerometer signal: A1, A2, A3, and A4. Their relationships to the cardiac mechanical activity, echocardiography, and ECG are displayed in Table 1. The post-ventricular atrial blanking period (PVAB) (500–550 ms by default), an interval that is used to blank the A1 and A2 signals, is followed by A3 and A4 sensing windows. The A3 window end (750–900 ms by default) is marked as “VE” annotation, denoting the end of all ventricular activity on the programmer. The A4 window starts at VE and ends with a ventricular sensed or paced event. The A3 threshold must be programmed higher than the A3 signal to blank the A3 signal, as opposed to the A4 threshold, which needs to be programmed lower than the A4 signal to sense A4. Any mechanical activity sensed during the A3/A4 window is denoted as AM. Any ventricular pacing is denoted as VP, and any ventricular activity sensed during AM-VP interval (20 ms by default) is denoted as VS. The device is programmed to accurately discern the A4 signal; that is, to accurately mark AM on the A4 signal so as to synchronize with VP [15,71,72,73] (Figure 2).

### 3.2. Evaluation of the AV Synchrony and Recommendation of Strategies

**Figure 3:**
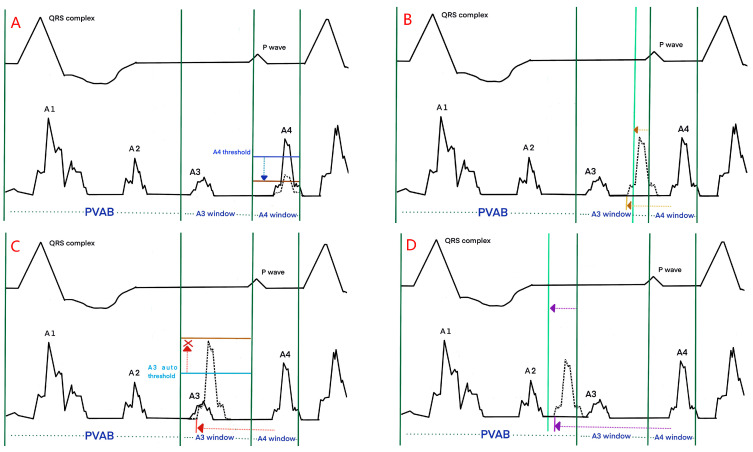
Schematic illustration and device programming in various situations. The blue dotted line indicates a lower A4 threshold in the situation of low A4 amplitude (**A**). The yellow dotted line indicates a shorter A3 window end interval in the situation of the A4 signal falling in the A3 window (**B**). The red dotted line indicates the deactivation of the A3 auto threshold function in the situation of the A4 signal merging with the A3 signal (**C**). The purple dotted line indicates a shorter PVAB in the situation of the A4 signal falling in the PVAB period (**D**).

The efficacy of the Micra AV was mainly evaluated based on the rate of AV synchrony. The median AV synchrony was 87% in the Micra Atrial TRacking using a Ventricular AccELerometer (MARVEL) study [15], and due to an enhanced algorithm, which included new features such as automated programming and mode switching, the median AV synchrony increased to 89.2% in the MARVEL2 study [16]. The average AV synchrony was 94.4% in patients with intrinsic conduction, compared with 80.0% in patients with high-degree AV blocks [15]. Patients with intrinsic conduction could have higher AV synchrony; however, the high-degree AV block with relatively low AV synchrony was the arrhythmia that the Micra AV was designed to treat. The AV synchrony in the MARVEL and MARVEL2 studies was objectively and accurately calculated with a Holter monitor; however, the processes were time-consuming. A currently published study in which the authors evaluated the relationship between AV synchrony and LPM device counters showed that the median AV synchrony of 87.1% was well correlated with a median %AM-VP of 79.1% in the MARVEL2 study [68], providing evidence for the reliability of the LPM device counters. Moreover, a linear and positive correlation between the AV synchrony determined with the Holter monitor and the LPM device counters was confirmed in another study [74]. In a real-world setting, the median %AM-VP was 74.7% among 1662 patients with %VP > 90% [68]. A highly similar ambulatory AV synchrony of 74.8%, assessed using Holter monitoring, was found in patients with complete AV blocks [75]. A theoretical AV synchrony of theoretical 100% is what clinicians and technicians expect and pursue. Factors that could influence the AV synchrony and the according strategies are listed as follows:

A. Patients’ factors:
  (a) Patient characteristics: High AV synchrony was associated with a lower BMI, a lower proportion of congestive heart failure, a history of cardiac surgery, and pulmonary hypertension [71]. It is a hypothesis the A4 amplitude was negatively related to a history of coronary artery bypass grafting due to the ischemia-inducing reduced atrial contractions [76];
  (b) Electrocardiogram (ECG): Some cases of low AV synchrony could be related to sinus rates < 50/min [15], and an analysis in a real-world setting of outpatients indicated that the median AV synchrony was 91% when the patients had sinus rates of 50–80/min, and that it decreased to 33% when the patients had sinus rates of >80/min [72]. Therefore, a sinus rate of 50–80/min contributes to high AV synchrony. Several kinds of arrhythmia, such as a sinus rate variability of >5 bpm at rest [76], AF/atrial flutter [71], and a high premature atrial/ventricular complex [15], are associated with lower AV synchrony. The A4 amplitude was positively correlated with *p*-wave amplitude in lead aVR [76];
  (c) Echocardiography: A higher A wave in the echocardiography reflects a stronger atrial contraction and a greater possibility of being discerned by the Mica AV. In a past study, the authors demonstrated that an E/A ratio of <0.94 indicated a high AV synchrony [76], in comparison with an E/A ratio of >1.5, which is considered a contraindication to Micra AV implantation. A small-sample study indicated that an A wave velocity > 73 cm/s could predict appropriate atrial sensing [77];
  (d) Maneuver and posture: The AV synchrony ranged from 89.2% during resting to 69.8% during standing, and to 74.7% during fast walking [16]. The higher sinus rate and volatile direction of the acceleration during activity could influence the sensing of atrial mechanical contraction, as reflected by the lower ambulatory AV synchrony of 74.7% a real-world setting [68], compared with that of 80.0% in a clinical trial for patients with AV blocks [15]. Hence, the Micra AV is more suitable for patients with sedentary lifestyles.
B. Procedure technique: The Micra AV implant location has not been reported to have a significant influence on the AV synchrony [16] or A4 amplitude [76]. In terms of the implant location selection, physicians should take the electrical parameters of the RV and relevant complications into consideration; however, the AV synchrony cannot be evaluated or mediated during the procedure, which is another drawback of the Mica AV hardware design.
C. Device programming: The nominal values of the Micra AV were optimized for patients during resting. Regular postimplant device reprogramming is necessary and should be individually optimized. The manual atrial mechanical (MAM) test is to line up A1–A4 signals with the corresponding surface ECG signals (Figure 2). Firstly, the MAM test with “auto” atrial mechanical features turned-off runs in the VDI mode to allow a clear distinction of the A1–A4 signals, and subsequently, MAM test runs in the VDD mode to make adjustment based on the track of atrial activity. The systematic and stepwise approaches including MAM test and adjustments of the A4 threshold, A3 window, and A3 threshold are to accurately discern A4 [78,79].
  a. The A4 threshold: In situations of low A4 amplitudes, a lower A4 threshold facilitates a reduction in the under-sensed A4 and improves the AV synchrony [72,73]; Meanwhile, in the case of low A4 amplitudes, the device’s built-in 3-axis accelerometer atrial-sensing vectors can be changed from a selection of one or two vectors to a recruitment of all three vectors to improve AV synchrony at the cost of negative impact on battery longevity [78,79]. When the A4 threshold is too low, the over-sensed A4 could impair the AV synchrony, which was observed in a study in which a higher A4 threshold was found to be related to a higher AV synchrony [74] (Figure 3A);
  b. The A3 window end: In situations of sinus tachycardia, the A4 signal falls in the A3 window, which reduces the AV synchrony. A shorter A3 window end interval for detecting the A4 signal and improving the AV synchrony has been confirmed in multiple studies [72,73,74]. A rate-dependent A3 window may be promising for tracking atrial contractions at higher heart rates. However, some researchers have suggested setting the A3 window below 700 ms and deactivating the automatic adjustment to improve the AV synchrony [74] (Figure 3B);
  c. The A3 threshold: In situations of sinus tachycardia, the A4 signal begins with the encroachment into the A3 window; however, as the heart rate is further elevated, the A4 signal could merge with the A3 signal and the A3 auto threshold function could result in the under-sensing of A4. Turning the A3 auto threshold function off and fixing the A3 threshold contribute to AV synchrony, and this is especially suitable for elevated sinus rates of 80–110/min [71]. A lower A3 threshold could improve the AV synchrony [74,76] (Figure 3C);
  d. The PVAB: In situations of Wenckebach behavior, the progressive shortening of the RP interval means that the P wave falls in the PVAB period, which results in the intermittent loss of A4 [76]. Shortening the PVAB to minimize the *p*-wave blanking is recommended. Wenckebach behavior occurs in patients with intrinsic conduction for whom the AV synchrony is high; therefore, the benefit of shortening PVAB is limited (Figure 3D);
  e. AV conduction mode switch: The algorithm of the Micra AV assumes intact intrinsic conduction in cases of ventricular rates of >40/min by default, and it switches to VVI-40 and VVIR pacing [16] if this function is activated. However, in situations of 2:1 AV blocks with sinus rates of ≥80/min, or complete AV blocks with ventricular escape beats of ≥40/min, such a function decreases the AV synchrony, and the recommendation is to switch it off [73,80];
  f. Rate smoothing feature: This feature was delivered at a rate smoothing interval (typically 100 ms) longer than the median R-R interval if A4 was not detected and improved the AV synchrony by 9% [15]. In situations of high sinus rate variability or high/low sinus rates, the efficacy of such a feature is somewhat compromised. Some studies suggested programming the rate smoothing interval longer than 100ms in patients with high sinus rate variabilities and low sinus rates [68], and programming the interval to 50 ms in patients with sinus rates of >90/min [16];
  g. Programmed lower rate: The loss of AV synchrony could be induced by sinus rates lower than the programmed lower rate [68,72]. The programmed lower rate should be set according to the sinus rate, measured using 24 h Holter monitoring.

## 4. Selection Strategy for LPMs vs. TPMs

### 4.1. Selection Strategy for VVI-LPMs vs. DDD-TPMs

A total of 36–38% of patients with VVI-LPMs had non-AF bradyarrhythmias [20,21], in which the absence of AV synchrony due to chronic VVI pacing could theoretically cause a decreased stroke volume and increase the incident AF and heart failure. However, only 1.1% of patients experienced heart failure and pacemaker syndrome related to the VVI pacing mode [81]. Another LPM registry from Italy found that patients without AF did not experience significantly higher rates of recurrent syncope, cardiac hospitalization, or all-cause death compared with patients with AF during a mean follow-up period of more than 600 days [82], which suggests that the effect of VVI-LPMs on patients with non-AF is reassuring. It remains to be seen whether non-AF patients will benefit more from DDD-TPMs than VVI-LPMs. A propensity-matched analysis indicated that VVI-LPMs for non-AF bradyarrhythmias significantly increased the rate of heart-failure-related rehospitalization at the 48 month follow-up compared with the use of DDD-TPMs, and a higher but not significant all-cause mortality was observed in patients with VVI-LPMs [63].

### 4.2. Selection Strategy for VDD-LPMs vs. DDD-TPMs

In addition to the lack of atrial pacing to treat sick sinus syndrome, the unachievable 100% AV synchrony, especially in cases of high sinus rates, is another shortcoming of the hardware design of the Micra AV. Thus, VDD-LPMs are the interim solution prior to the realization of true DDD-LPMs. Although the AV synchrony substantially decreased at sinus rates of >80/min [72], the cardiac output at higher rates has been shown to be more dependent on the heart rate than the AV synchrony [83]. A positive chronotropic response provides approximately 75% of the increment in cardiac output, whereas the maintenance of AV synchrony and increased contractility accounting for the remaining 25% [84]. Therefore, rate responsive pacing could be an adequate option for patients when exercising. As for sedentary or elderly patients with AV blocks, compared with DDD-TPMs the Micra AV might still have the advantage, despite the less than 100% AV synchrony. However, there is an argument that the benefit of AV synchrony is not essential at rest, as the pacemaker syndrome causes symptoms, especially during exercise. Therefore, whether the added value of the Micra AV vs. Micra VR in clinical practice could be evaluated with clinical endpoints needs further exploration.

### 4.3. Conduction System Pacing

The RV apical pacing and RV septal pacing result in dyssynchronous activation that can impair ventricular function and have the possibility of inducing pacing-induced cardiomyopathy [85]. Conduction system pacing, including His bundle pacing and left bundle branch pacing, has gained prominence as a novel pacing modality that can activate the ventricles physiologically, correct bundle branch block, and improve cardiac function via the lead of TPMs fixed in the specific region of the His–Purkinje conduction system [86]. Improvement in the LPM hardware design, such as a more precise fixation mechanism and contact mapping capability of the Aveir VR, can perhaps provide a promise of fulfilling conduction system pacing by LPMs.

## 5. Leadless Atrial Pacemakers

The Atrial Micra (Medtronic) and Aveir atrial LPM (Abbott) are two kinds of LPMs that are positioned in the RA appendage and that are designed to deliver the AAI(R) mode. The two have been tested and evaluated in preclinical ovine studies. The Atrial Micra is a modified version of the Micra VR, featuring shorter and flatter tines that are adaptive for relatively thin atrial myocardium [87]. The Aveir atrial LPM is also a modified version of the Aveir VR, with a dual-helix fixation mechanism that is specific to the RA anatomy [88], and with an electrically active inner helix and mechanical fixation of the outer helix. The preliminary results indicated that both of these AAI-LPMs exhibited excellent and stable pacing performances during the 24 week follow-up for the Atrial Micra and the 12 week follow-up for the Aveir atrial LPM. Here, the Atrial Micra displayed its potential to be retrieved. As for safety concerns, one dislodgment of the Atrial Micra occurred among 17 implants and one case of hemopericardium occurred during the retrieval and implant procedure. In comparison, no dislodgments or significant myocardial perforations of the Aveir atrial LPM have been reported. Phase III clinical trials are expected to further assess the efficacy and safety of these two AAI-LPMs.

## 6. Dual-Chamber Leadless and Battery-Less Pacemakers

### 6.1. The Conception of True Dual-Chamber Leadless and Battery-Less Pacemakers

The configuration of a true DDD-LPM is conceptualized as the combination of a VVI-LPM with an AAI-LPM, and it is supported by the device–device communication technology and algorithm to coordinate the atrium and ventricle activities. An Aveir DR is under development by Abbott, which has a proprietary implant-to-implant communication technology for the regulation of AAI-LPMs and VVI-LPMs in a dual-chamber fashion [89]. Due to the limited volume of LPMs and complex environment of the beating heart, high energy efficiency, excellent interference resistance, and long-term reliability are the main requirements for the communication technology. The small form factor of the battery size constrains its long-term usage. In addition to the currently available battery technology under constant improvement, some novel energy programs are under development to address this quandary.

### 6.2. Communication Technology for the Realization of Dual-Chamber Leadless Pacemakers

#### 6.2.1. Radio-Frequency (RF) Communication

Unlike communications through the air, the various tissues and organs within the body have different conductivities, dielectric constants, and impedances, which could interfere with RF communication. A frequency range of 2.4–2.5 GHz in in vivo experiments is optimal for the multi-node pacemaker technology with the least signal attenuation [90]. The RF communication is prone to electromagnetic interference and suffers from large signal leakages and the ease of eavesdropping.

#### 6.2.2. Conductive Intracardiac Communication (CIC)

CIC, which is also known as galvanic coupled intra-body communication, is a promising novel technique for highly energy-efficient wireless communication inside the heart. The conduction of electrical signals between LPMs through the blood and myocardial tissue should be sufficient to convey information, and it is lower than the pacing threshold to avoid pacing myocardium. A frequency range of 100 kHz–1 MHz in in vivo experiments is beneficial [91]. Compared with RF communication, CIC has a lower system power consumption and electromagnetic interference, as well as higher data security, because the communication signal is confined within the human body. The trade-off for the low frequency and power consumption is the data rate; fortunately, the data rate required for the vital physiological signal transmission among implantable devices is relatively low. The dual-chamber pacing modes were tested unidirectionally, from the RA to RV and from left ventricle (LV) to the RV, through CIC, and specific algorithms have been tested in vivo in pigs [92]. The chronic preclinical feasibility of two LPMs using the novel bidirectional communication technology was evaluated in ovine subjects [93], and the stable performance of the bidirectional communication during the 13 week follow-up and the energy efficient algorithm of the communication modality were demonstrated.

### 6.3. Energy Programs for the Realization of Dual-Chamber Battery-Less Pacemakers

#### 6.3.1. Acoustic Energy

The wireless Stimulation Endocardially for CRT (WiSE-CRT) system (EBR Systems, Sunnyvale, CA, USA) was developed to emit ultrasonic energy from a subcutaneous ultrasound transmitter to a wireless LV endocardial receiver electrode, which paces the LV by converting the ultrasonic energy into electrical energy [94]. Once the battery is depleted, changing the subcutaneous battery is sufficient without the need for removing the endocardial electrode. One disadvantage is that 7.7% of patients did not have an adequate acoustic window to power the receiver electrode [95], and another is the low energy efficiency of acoustic energy [96]. A narrowed ultrasound beam could increase the energy efficiency; however, it would be susceptible to the change in the beam location and posture [97]. Therefore, one subcutaneous transmitter to drive multi-site receivers is more difficult under the constraint of the acoustic window.

#### 6.3.2. RF Energy

An intravenous cardiac pacemaker designed to be implanted in cardiac veins is a passive wireless power receiver circuit that receives bursts of power at 13.5 MHz from a subcutaneous transmitter and stimulates the tissue [98]. A novel bioresorbable leadless cardiac pacemaker for the purpose of temporary pacing is powered by a wireless inductive energy transfer at a frequency of 13.5 MHz [99]. The adoption of 13.5 MHz is because there is less absorption by biofluids or biological tissues at this frequency regime [99]. The advantage of RF energy is its potential to be extended to multi-chamber pacing by using different frequencies.

#### 6.3.3. Kinetic Energy

The kinetic energy derived from cardiac vibrations [100,101], blood flow [102] and body motion [103] could serve as an inexhaustible source for battery-less pacemakers. A mass imbalance or oscillation weight is connected to an electrical micro generator to convert a minimal amount of the heart’s kinetic energy into electric energy [100,101]. Due to the nature of kinetic energy, the selection of the implantation site should consider the direction of the cardiac contraction or blood flow. The relatively low efficiency of in vivo energy harvest is another drawback of kinetic energy. A novel coin battery-sized inertia-driven triboelectric nanogenerator that is dependent on the body motion and gravity to produce electricity demonstrated a significant power performance in preclinical settings [103].

## 7. Conclusions

Although LPMs have shown excellent and stable pacing performances and are associated with a low rate of complications in comparison with TPMs, some risk complications, such as cardiac perforation and dislodgment, should be handled with extra caution and individualized strategies. Compared with LPMs, DDD-TPMs could bring about lower rates of heart-failure-related rehospitalizations for patients with non-AF; however, clinicians should balance the long-term benefits of the low complication rates of VVI-LPMs with the benefits of AV synchrony and/or interventricular synchrony of DDD-TPMs prior to making clinical recommendations. With the further development of AAI-LPMs, true DDD-LPMs could theoretically realize almost all the features of DDD-TPMs, except for the conduction system pacing; however, DDD-LPMs inevitably increase the risk of atrial perforation and dislodgment due to the extra AAI-LPMs. Hence, VDD-LPMs still have their own advantages for patients with sedentary lifestyles and at high risk of pericardial effusion. The prospect of LPMs is promising and encouraging. The integration of leadless pacing and conduction system pacing, more efficient and reliable communication technology, and improved battery technology or, alternatively, revolutionized energy programs, require further exploration.

## Figures and Tables

**Table 1:** Meanings of A1–A4 in relation to function, echocardiography, and electrocardiogram.

		Heart Sound	Echocardiography	Electrocardiogram
A1	Mitral/tricuspid valve closure	S1		At the end of QRS complex
A2	Aortic/pulmonic valve closure	S2		At the end of T wave
A3	Early passive ventricular filling	S3	E wave	Before the onset of the P wave
A4	Atrial contraction	S4	A wave	After the inscription of the P wave

## Data Availability

Not applicable.
